# Study on synergistic effects of 4f levels of erbium and black phosphorus/SnNb_2_O_6_ heterostructure catalysts by multiple spectroscopic analysis techniques†

**DOI:** 10.1039/d3sc05464k

**Published:** 2024-01-05

**Authors:** Minze Li, Jingzhen Wang, Qiuye Wang, Honglai Lu, Guofeng Wang, Honggang Fu

**Affiliations:** a Key Laboratory of Functional Inorganic Material Chemistry, Ministry of Education, School of Chemistry and Materials Science, Heilongjiang University Harbin 150080 China 2010070@hlju.edu.cn fuhg@vip.sina.com

## Abstract

Lanthanide single atom modified catalysts are rarely reported because the roles of lanthanide in photocatalysis are difficult to explain clearly. Based on the construction of Er single atom modified black phosphorus/SnNb_2_O_6_ (BP/SNO) heterojunctions, the synergistic effect of 4f levels of Er and heterostructures was studied by combining steady-state, transient, and ultrafast spectral analysis techniques with DFT theoretical calculations. According to the Judd–Ofelt theory of lanthanide ions, the CO_2_ photoreduction test under single wavelength excitation verifies that the ^4^F_7/2_/^2^H_11/2_ → ^4^I_15/2_ emissions of Er in BPEr/SNOEr can be more easily absorbed by SNO and BP, further proving the role of the 4f levels. As a result, the CO and CH_4_ yields of BPEr/SNOEr-10 under visible light irradiation are 10.7 and 10.1 times higher than those of pure BP, respectively, and 3.4 and 1.5 times higher than those of SNO. The results of DFT calculations show that the Er single atoms can cause surface reconstruction, regulate the active sites of BP, and reduce the energy change value in the key steps (CO_2_* + H^+^ + e^−^ → COOH* and COOH* → CO* + H_2_O). This work provides novel insights into the design of lanthanide single atom photocatalysts for CO_2_ reduction.

## Abstract 2

Visible-light-driven CO_2_ photoreduction performance can be improved by utilizing the synergistic effect of BPEr/SNOEr heterostructures, Er single atoms and the Er 4f energy level to facilitate light absorption.

## Graphical abstract

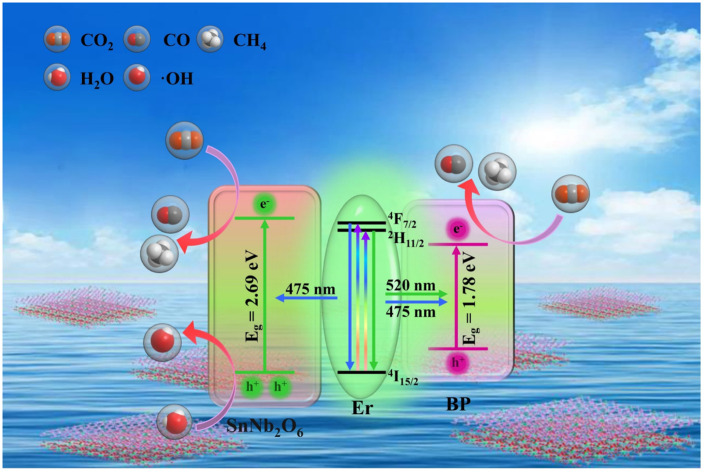

## Introduction

In the era of rapid development of industrialization, energy and the environment have already become two main challenges that human beings have to face. The greenhouse effect caused by the massive emission of CO_2_ poses a serious threat to the global ecosystem, so it is imperative to reduce CO_2_.^1–6^ The reduction of CO_2_ to usable chemicals driven by solar energy is a promising strategy for the elimination of greenhouse gases and the provision of renewable energy.^7–12^ However, the developed photocatalysts still have some limitations such as a narrow photo-absorption range and low photocarrier separation efficiency, resulting in low photocatalytic efficiency.

Compared with traditional photocatalysts, single-atom catalysts (SACs) can enhance the photocatalytic activity, because SACs can expose more active sites and have higher atom utilization efficiency.^13–17^ Among the SACs, rare earth (RE) single atom photocatalysts are rarely reported. Specifically, it is still a great challenge to reveal the mechanism of how RE single atoms improve the performance of photocatalysts due to the complexity of the electronic layer structure of the RE ions, which limits the development and utilization of RE SACs. In recent years, our research group has carried out some preliminary exploration of RE SACs, but the research on the photocatalyst mechanism is not comprehensive or in-depth enough.^18,19^

It is well known that SACs are synthesized by anchoring isolated metal atoms onto solid matrix materials. Among the catalyst materials reported, SnNb_2_O_6_ (SNO) is a typical 2D semiconductor photocatalyst with many unique advantages, such as a suitable band gap (about 2.60 eV), large specific surface area, more active sites, and high stability.^20–22^ Nevertheless, the photocatalytic activity of the SNO nanosheets is usually limited by the rapid recombination of photogenerated carriers within the material.^23–25^ It is well known that the construction of composite systems by using catalysts with unequal band structures is one of the effective strategies to achieve efficient photoconversion of CO_2_.^26–28^ Therefore, the modification of SNO by ion doping or constructing a composite system is an effective method to improve the photocatalytic ability.^29–31^ So far, there are no reports about RE single atom modified SNO or SNO-based heterostructures.

Black phosphorus (BP) is a graphene-liked layered material, which also shows tremendous potential in the field of photocatalysis owing to its preeminent physical and chemical properties.^32–38^ BP shows an adjustable band gap ranging from 0.3 to 2.0 eV, which depends on its change from bulk to monolayer. Furthermore, monolayer BP exhibits the highest charge carrier migration rates of up to 10^3^ cm^2^ V^−1^ s^−1^ at room temperature and broad solar light absorption.^39^ More importantly, BP is a very good RE single atom carrier material.^18^ However, the photocatalytic performance of unmodified BP is still very low.^40,41^ To overcome this shortcoming, the use of BP and other semiconductors to construct heterostructure systems has proved to be an effective strategy. We suggest that if BP and SNO are combined to form heterojunctions, and then the heterojunction is further modified by using RE single atoms, unexpected properties may be obtained by using the synergistic effects of RE single atoms and BP/SNO heterojunctions.

Here, the work focused on the synergistic effects of heterostructures and Er single atoms, constructed BPEr/SNOEr heterostructures, and finally achieved the goal of improving the photocatalytic performance. The results indicated that the Er single atom modified heterojunction showed the highest photocatalytic activity, which is not only because the introduction of BP has played multiple roles, but also because the Er single atoms can cause surface reconstruction, adjust the active site of BP, and reduce the energy change value in the key steps (CO_2_* + H^+^ + e^−^ → COOH* and COOH* → CO* + H_2_O) of the photocatalytic CO_2_ reduction process. More importantly, the ^4^F_7/2_/^2^H_11/2_ → ^4^I_15/2_ emissions of Er ions can be further absorbed by BP/SNO to promote the light absorption and improve the photocatalytic efficiency based on the J–O theory of RE ions as well as the CO_2_ photoreduction test under a single wavelength excitation.

## Results and discussion

**Fig. 1:**
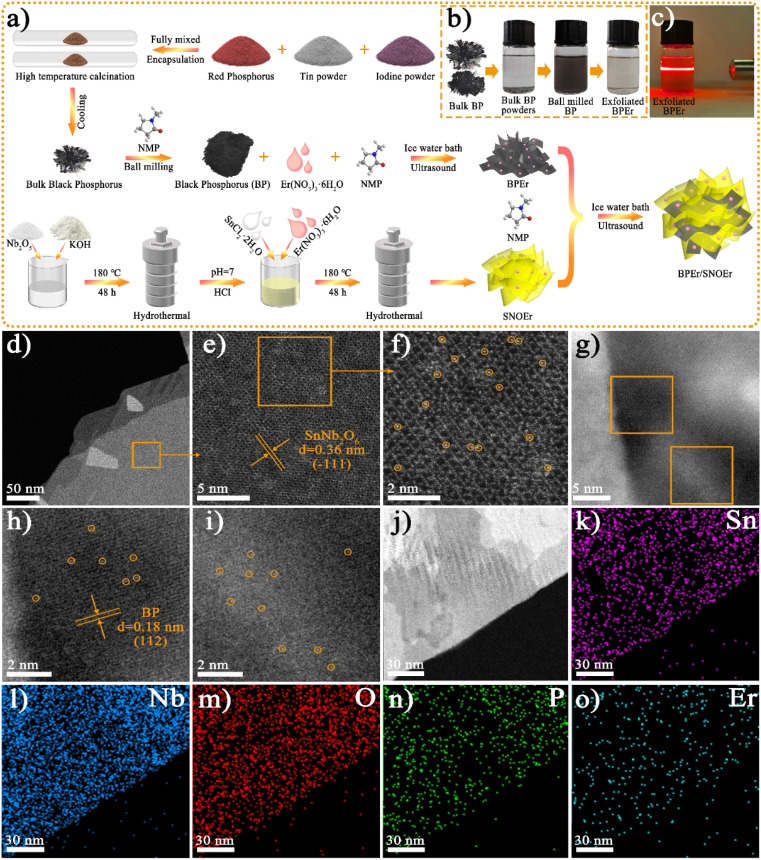
(a) Schematic diagram of the catalyst preparation process. (b) Photos of different states of BP and BPEr. (c) Photo of the Tyndall effect of BPEr. (d–i) AC HAADF-STEM images of BPEr/SNOEr-10. (j–o) HAADF-STEM image and EDX elemental mappings of BPEr/SNOEr-10.

The synthesis procedures and sample abbreviations of different samples are illustrated in Fig. 1a and Tables S1–S2.† The characterization of materials, the photocatalytic CO_2_ reduction performance, and the synergistic effects of heterojunctions and Er single atoms on photocatalytic performance were studied through experimental and DFT theory calculations.

### Material characterization

The photos of BP in different states show that the ball-milled BP and the exfoliated BPEr have good dispersion in NMP solution, and the exfoliated BPEr exhibits the Tyndall effect (Fig. 1b and c). The scanning electron microscope (SEM) images indicate that SNO and SNOEr nanosheets clearly exhibit a smooth, ultrathin, and layered stacked structure, as shown in Fig. S1.† The morphology of SNO has no obvious change after RE ion doping. For BP/SNOEr-5, it can be observed that the ultrathin SNOEr sheets are attached to the surface of BP sheets. However, it is difficult to detect the presence of BP when the proportion of BP decreases to a certain extent. The transmission electron microscope (TEM) and high-resolution TEM (HRTEM) images further show the lamellar structure and lattice fringes of SNO and BP (Fig. S2†). The crystal plane spacings of 0.28 and 0.26 nm are attributed to the (600) plane of SNO and the (040) plane of BP, respectively. The lattice stripes of both BP and SNO can be clearly seen in the HRTEM images of BPEr/SNOEr-10, which can well correspond to the above crystal planes. The energy-dispersive X-ray spectroscopy (EDX) elemental mappings in the corresponding TEM area show that Sn, Nb, O, P, and Er elements are uniformly distributed in the composite, which further confirms that the BPEr/SNOEr-10 heterojunction has been successfully constructed (Fig. S3†).

**Fig. 2:**
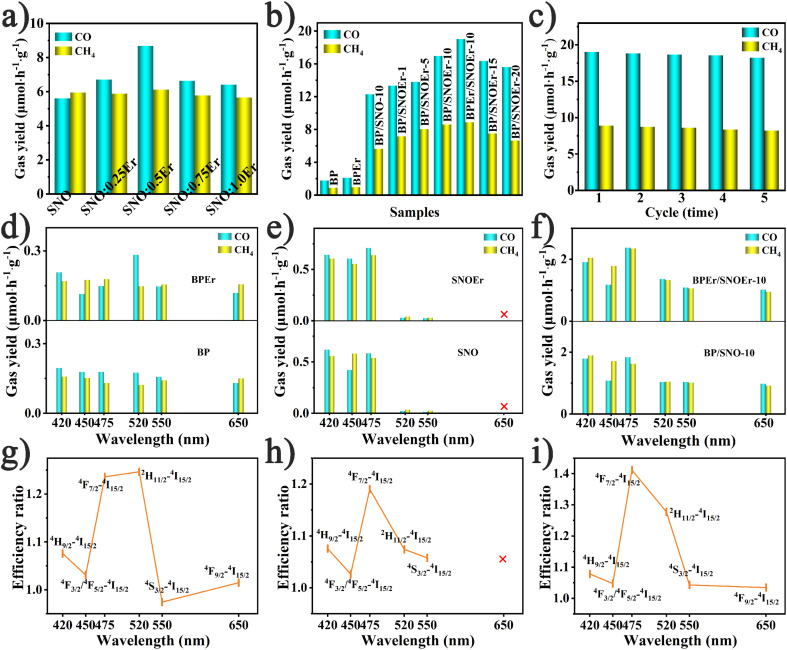
(a and b) CO_2_ photoreduction production rates of SNO:*x*Er, BP, BPEr, BP/SNO, BP/SNOEr, and BPEr/SNOEr. (c) Stability of the BPEr/SNOEr-10 photocatalyst. (d–f) Production rates of BP, BPEr, SNO, SNOEr, BP/SNO-10, and BPEr/SNOEr-10 under different single wavelength excitation. (g–i) The efficiency ratios of samples containing Er and samples without Er.

In order to verify the states of Er in composites, aberration-corrected high-angle annular dark-field scanning transmission electron microscopy (AC HAADF-STEM) and STEM-EDX were performed (Fig. 2). The (−111) crystal plane of SNO is deduced from the well resolved lattice fringes of AC HAADF-STEM (Fig. 2e), and isolated bright points highlighted by yellow circles are identified as single Er atoms (Fig. 2f). Moreover, the Er single atoms were also observed on the surface of BP (Fig. 2g–i). In addition, the STEM-EDX elemental mappings also clearly show that Sn, Nb, O, P, and Er are distributed evenly on the entire framework (Fig. 2j–o).

The X-ray diffraction (XRD) results show that SNO can be easily assigned to the monoclinic phase (JCPDS: 84-1810), and the diffraction peaks are sharp and strong, indicating that it is highly crystalline (Fig. S4†). No obvious diffraction peak of RE ions was observed in the samples doped with RE ions, indicating that the atomic-level dopants of RE ions were highly dispersed on SNO, which was consistent with the results of HAADF. For BP/SNOEr, the characteristic peaks of BP (JCPDS: 73-1358) and SNOEr are detected as expected, which proves that the BP/SNOEr heterojunction is composed of BP and SNOEr. The results of energy dispersive spectroscopy (EDS) further prove that Sn, Nb, O, P, and Er elements exist in BPEr/SNOEr-10 (Fig. S4c†). The results of Fourier transform infrared spectroscopy (FT-IR) indicate that the peak at ∼667 cm^−1^ is assigned to the representative vibration mode of Nb–O in the NbO_6_ octahedron, and the peaks at ∼1010 and ∼3400 cm^−1^ are assigned to the P–O–P and O–H stretching vibration modes (Fig. S4d†), respectively. The results of ICP-MS show that the mass concentration of Er in BPEr/SNOEr-10 is 0.05%.

The absorption edge of SNO is observed in the UV-visible diffuse reflectance spectrum (DRS), and the doping of Er makes the absorption edge of SNO red shift, indicating that SNOEr has an enhanced visible light response (Fig. S4e†). For the composite samples, the introduction of BP makes the absorption range red shift to ∼800 nm, which is conducive to improving the photocatalytic performance. The bandgap values of SNO, SNOEr, BP, and BPEr are 2.69, 2.63, 1.78, and 1.54 eV, respectively (Fig. S4f†). The valence band (VB) and conduction band (CB) positions of SNO and BP are obtained from the XPS valence band spectra (Fig. S4g†) and the Mott–Schottky curves (Fig. S4h and I†), respectively. The detailed calculation method is described in the ESI.†

The composition and interaction of BPEr and SNOEr in heterojunctions were further analyzed by X-ray photoelectron spectroscopy (XPS, Fig. S5 and S6†). The peaks of SNO at 486.17 and 496.62 eV, 206.88 and 209.65 eV, and 529.93 and 532.18 eV are assigned to Sn 3d_5/2_ and 3d_3/2_, Nb 3d_5/2_ and 3d_3/2_, and O 1s lattice oxygen and adsorbed oxygen, respectively. For the spectra of BP, the two bands at 129.66 and 130.56 eV can be attributed to P 2p_3/2_ and 2p_1/2_, respectively. The peak at 133.78 eV originates from oxidized phosphorus (P_*x*_O_*y*_) produced during the preparation of BP. Furthermore, the peaks at 531.40 and 532.92 eV in the O 1s spectra of BP corresponded to P–O and P–OH, respectively. For the spectra of SNOEr and BPEr samples, the binding energies of all elements shifted after Er doping, which may be due to the strong interaction between the Er single atoms and the catalyst. Similarly, compared with single-component SNOEr and BPEr, the peak positions of BPEr/SNOEr-10 also shift. Among several elements, Sn 3d and P 2p exhibit more obvious binding energy shifts. Compared with SNOEr, the peak positions of Sn 3d of BPEr/SNOEr-10 shift to the high binding energy direction. Compared with BPEr, the peak positions of P 2p shift to the low binding energy direction, demonstrating that SNOEr strongly interacted with BPEr and the charge is transferred from SNOEr to BPEr. More importantly, a new strong peak at 139.22 eV attributed to P–O was detected in the spectrogram of P 2p, indicating that BP can take O as well as form O vacancies on the SNOEr surface, which can be further proved by the electron paramagnetic resonance (EPR) results later.

### CO_2_ photoreduction performance and spectroscopic analysis

The CO_2_ photoreduction performance measurements were carried out in pure water without any sacrificial agent. It is noted that all the photocatalyst properties of BP, SNO, and BP/SNO were improved by the modification using Er ions. The yields of CO and CH_4_ for SNO are 5.60 and 5.96 μmol h^−1^ g^−1^, respectively. The introduction of different RE ions has different effects on the performance of SNO (Fig. S7 and Table S1†). Among all the as-prepared RE ion doped catalysts, SNOEr shows the highest CO (8.68 μmol h^−1^ g^−1^) and CH_4_ (6.12 μmol h^−1^ g^−1^) yields. Among all SNO:*x*Er (*x* = 0.25, 0.5, 0.75 and 1.0), SNO:0.5Er (SNOEr) has best photocatalytic activity (Fig. 2a). For the BP/SNOEr composite photocatalyst with different BP contents, BP/SNOEr-10 showed the highest CO and CH_4_ production rates (Fig. 2b). In addition, the photocatalytic performance of BPEr/SNOEr-10 obtained by compounding BPEr with SNOEr is better than that of BP/SNOEr-10. For BPEr/SNOEr-10, the CO yield (19.01 μmol h^−1^ g^−1^) is 3.4 and 10.7 times that of pure SNO and BP, and the CH_4_ yield (8.89 μmol h^−1^ g^−1^) is 1.5 times and 10.1 times that of pure SNO and BP, respectively. In these reactions, no H_2_ product was detected and the oxidation product was O_2_ (Fig. S8†). It is noted that the oxidation reaction is the oxidation of H_2_O by holes to O_2_ and H^+^. These H^+^ were not completely consumed to produce CH_4_ during the 8-electron process, and some of them participated in the reaction to produce CO during the 2-electron process. The specific discussions can also be found in the ESI† section. In addition, the selectivity of CO and CH_4_ is 34.8% and 65.2%, respectively. After 5 cycles of experiments, it was found that the CO_2_ photoreduction capacity of BPEr/SNOEr-10 was only slightly reduced, indicating that BPEr/SNOEr-10 was stable and reusable (Fig. 2c). The XRD patterns of BPEr/SNOEr-10 before and after the photocatalytic reaction have no obvious change, which also proves its excellent stability (Fig. S9†).

To study the influence of emission light generated by the transitions between different energy levels of Er ions on the photocatalytic performance, CO_2_ photoreduction measurements were carried out with 420, 450, 475, 520, 550, and 650 nm bandpass filters (Fig. 2d–f). It is noted that these wavelengths correspond to the transitions from the ^2^H_9/2_/^4^F_3/2_(^4^F_5/2_)/^4^F_7/2_/^2^H_11/2_/^4^S_3/2_/^4^F_9/2_ levels to the ^4^I_15/2_ level, respectively. The efficiency ratio refers to the photocatalytic efficiency ratio between catalysts with and without Er single atom anchoring under the corresponding single wavelength light excitation (Fig. 2g–i). Specifically, the efficiency ratios in Fig. 2g–i are calculated based on the efficiencies in Fig. 2d–f, respectively, and the calculation method of the efficiency ratio is listed in the ESI.† The cross in Fig. 2e and h shows that the efficiency ratio does not exist. It is noted that SNO and SNOEr do not have light absorption ability at 650 nm, so the yield of CO and CH_4_ is zero, and the efficiency ratio does not exist. According to the transition selection rules of RE ions, the probability of radiative and non-radiative transitions between different energy levels is different due to the different electronic layer structures of the different RE ions, resulting in different RE ions having different effects on photocatalysis. According to J–O theory, the probability of radiation transition between the energy levels of RE ions is determined by using the following formula.1
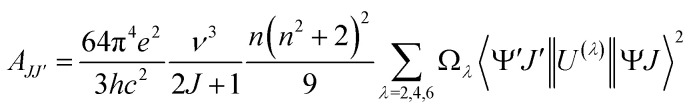
Here, *e*, *ν*, *n*, *h*, *c*, and 〈*Ψ*′*J*′*||U*^(*λ*)^*||ΨJ*〉^2^ are the elementary charge, mean wavenumber, refractive index, Planck constant, velocity of light in a vacuum, and doubly reduced matrix elements, respectively.^42^ Generally, the *U*^(*λ*)^ values between different energy levels of different RE ions are regarded as constant, and the *Ω*_*λ*_ values are related to the matrix materials. Here, the results show that different Er levels have different effects on the improvement of photocatalytic performance. For BPEr, the ^4^F_7/2_/^2^H_11/2_ → ^4^I_15/2_ emissions can be more easily absorbed by BP, which improves the utilization of light and thus improves the photocatalytic efficiency. It is well known that probabilities of radiation transition of both ^4^F_7/2_ → ^4^I_15/2_ and ^2^H_11/2_ → ^4^I_15/2_ emissions are larger than those of other emissions of Er ions due to the small stimulated absorption (transition) probability of ^4^I_15/2_ → ^4^H_9/2_ and ^4^I_15/_ → ^4^F_3/2_/^4^F_5/2_ as well as the energy loss caused by effective ^4^H_9/2_ → ^4^F_3/2_/^4^F_5/2_ and ^4^F_3/2_/^4^F_5/_ → ^4^F_7/2_ non-radiation transitions. And thus, only the ^4^F_7/2_/^2^H_11/2_ → ^4^I_15/2_ emissions can be effectively absorbed by BP for the second time, thus improving the photocatalytic efficiency. For SNOEr, the ^4^F_7/2_ → ^4^I_15/2_ emission can be most easily absorbed by SNO, and thus, the efficiency of photocatalysis is improved most obviously under 475 nm excitation, which is also a normal phenomenon. Different matrix materials have different phonon energies, which directly affect the stimulated absorption (transition) as well as non-radiative transition processes of materials. We suggested that the stimulated absorption (transition) probability of ^4^I_15/2_ → ^4^H_9/2_, ^4^I_15/2_ → ^4^F_3/2_/^4^F_5/2_, and ^4^I_15/2_ → ^2^H_11/2_ is invalid compared to that of ^4^I_15/2_ → ^4^F_7/2_ in SNOEr, so the ^2^H_11/2_ → ^4^I_15/2_ emission is a bit weak, which cannot greatly improve the photocatalytic performance of SNOEr. Similarly, due to the influence of the energy level position, Ce and Dy ions are also suitable to improve the light absorption of the catalyst, but Gd and Eu cannot play such a role (Fig. S10 and S11†). The impact of radiative transitions on electron transfer can be found in the ESI† section.

It is well known that CO_2_ adsorption is a committed step in photoreduction. The CO_2_-TPD curves confirmed that BPEr/SNOEr-10 had a stronger CO_2_ adsorption capacity (Fig. S12†). As mentioned above, O vacancies are beneficial to the adsorption and activation of CO_2_ molecules on the surface of the photocatalyst.^43^ Compared with SNO, BP/SNO-10 showed an additional peak of oxygen vacancies at *g* = 2.001 in the EPR spectra, which is caused by the interaction between BP and SNO (Fig. S13†). Due to the influence of Er ions, SNOEr showed a wide signal peak, and the signal peak of the oxygen vacancies of BPEr/SNOEr-10 was also wider than that of BP/SNO-10 (Fig. S14†).

The EPR measurement was further carried out with DMPO as the trapping agent to identify the active radicals generated during the photo reaction (Fig. S15†). Under dark conditions, no signal peak was observed in the EPR spectra of SNOEr and BPEr/SNOEr-10. When the samples were irradiated with visible light for 10 minutes, the signal peak of ˙OH was observed, and the signal of BPEr/SNOEr-10 was significantly higher than that of SNOEr. Under visible light irradiation, BPEr/SNOEr-10 can generate more electrons and holes, which are separately conducive to the reduction of CO_2_ and water oxidation in a photocatalytic reaction. The same results were obtained by the coumarin fluorescence method (Fig. S16†). The highest fluorescence intensity of BPEr/SNOEr-10 proved that it produced the most ˙OH. The above results indicate that during the photocatalytic process, holes in the SNOEr VB can oxidize water to generate ˙OH, rather than transfer to the BP VB.

**Fig. 3:**
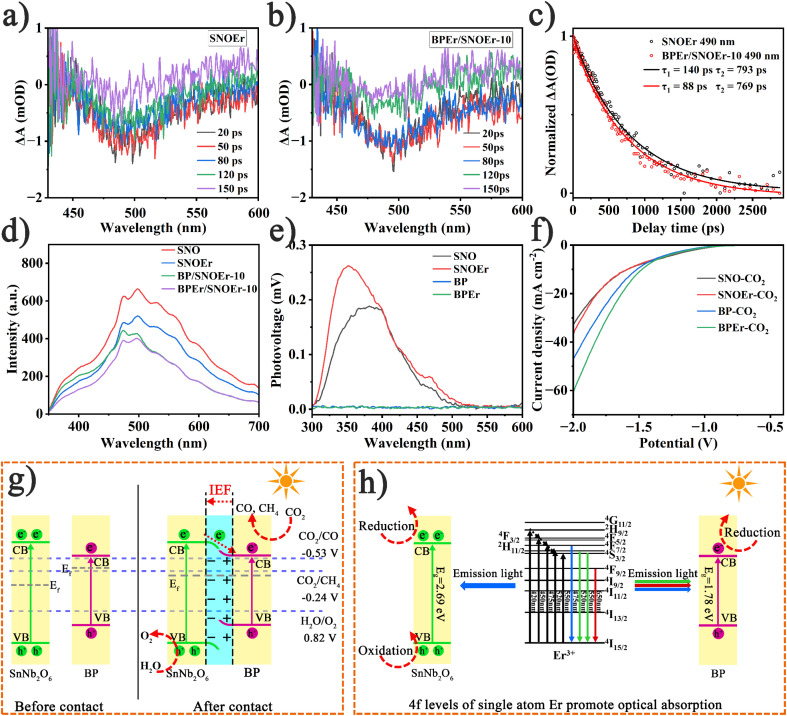
Femtosecond transient absorption spectroscopy (fs-TAS) (*λ*_ex_ = 325 nm) of (a) SNOEr and (b) BPEr/SNOEr-10. (c) Kinetics at 490 nm. (d) PL spectra (*λ*_ex_ = 325 nm) of different samples. (e) SPS responses of different samples. (f) Linear sweep voltammetry (LSV) curves of different samples in CO_2_-saturated KHCO_3_ (0.5 M) solution. (g) Energy band structure model and schematic diagram of the BPEr/SNOEr heterojunction. (h) Schematic diagram of Er assisting the composite photocatalyst to absorb light and thus improve the photocatalytic performance.

To further monitor the photogenerated carrier dynamics of the prepared catalysts, fs-TAS measurements were performed under 325 nm laser excitation (Fig. 3a and b). For SNOEr, there is significant negative band absorption at approximately 440 to 550 nm, with the maximum absorption peak at 490 nm. The spectral characteristics of BPEr/SNOEr-10 are similar to those of SNOEr. The absorption peak intensity of all samples decreases over time, indicating a decrease in active photogenerated charges. The dynamic attenuation curves of SNOEr and BPEr/SNOEr-10 were analyzed using the double exponential function (Fig. 3c). BPEr/SNOEr-10 has a shorter photogenerated carrier lifetime, which further proves the charge transfer from SNO to BP. It is noted that the electron transfer time from SNO to BP is at the the picosecond level, while the electron–hole recombination time of SNO is at the the microsecond level (Fig. S17†), and therefore, the construction of heterojunctions is conducive to charge transfer and suppresses charge recombination.^44^ In addition, the average lifetimes of samples modified with Er single atoms are obviously higher than that of pure samples, indicating that Er single atoms contribute to the separation of photogenerated charge carriers, thereby enhancing the photocatalytic activity of catalysts (Fig. S17†).

Usually, the photoluminescence (PL) intensity can also reflect the degree of recombination of photogenerated charge carriers, and the significant PL intensity is mainly due to the high recombination of photogenerated carriers. Under 325 nm light excitation, BPEr/SNOEr-10 displayed lower emission intensity in the PL spectra (Fig. 3d). For comparison, the PL spectra of BP and BPEr are also obained (Fig. S18†). In electrochemical testing, BPEr/SNOEr-10 showed the highest photocurrent density and the smallest arc radius in electrochemical measurements (Fig. S19 and S20†). These results show that BPEr/SNOEr-10 has excellent charge separation and transport capacity, which is consistent with the remarkable photocatalytic activity of BPEr/SNOEr-10, and it is an excellent photocatalyst.

The results of Hall effect testing indicated that the carrier concentrations of SNO, SNO:0.25Er, and SNO:0.5Er are 7.05 × 10^14^, 1.09 × 10^16^, and 3.14 × 10^16^ cm^−3^, respectively (Table S3†). Obviously, the introduction of Er single atoms increases the carrier concentration of the sample, which is consistent with the discussion above.

It is noted that the CO_2_ photoreduction performance of BP (or BPEr) is far inferior to that of SNO (or SNOEr). The results of surface photovoltage spectroscopy (SPS) indicated that the poor photocatalytic performance of BP and BPEr was caused by poor charge separation ability (Fig. 3e). To determine whether BP was not suitable as a catalyst, BP, BPEr, SNO, and SNOEr were used as catalysts to conduct the CO_2_ reduction test in the presence of an electric field. It is surprising that BPEr and BP have a better CO_2_ reduction performance than SNO and SNOEr (Fig. 3f and S21†). After the heterojunction is constructed, SNO can transfer electrons to BP, and BP plays a catalytic role. Therefore, the photocatalytic performance of the heterojunction has been greatly improved.

In order to investigate the charge transfer phenomenon during CO_2_ photoreduction, quasi *in situ* XPS measurements were conducted in a CO_2_ filled atmosphere (Fig. S22†). After the photocatalytic reaction, new peaks were surprisingly discovered in the spectra of Sn and Nb. These peaks can be attributed to metal peaks formed during the photocatalytic process due to the generation of a large number of oxygen vacancies and the absence of oxygen atoms in SNOEr. In the spectra of element P, the binding energies of P 2p_3/2_ and P 2p_1/2_ move in the direction of decrease, while in the spectra of element O, the binding energy of lattice oxygen moves in the direction of increase. This result shows that the charge is transferred from SNOEr to BPEr. In addition, the binding energy of Er shows a positive shift, which corresponds to the electron transfer between the catalyst and the adsorbed CO_2_ molecules in the reaction process.

A potential mechanism of the BPEr/SNOEr-10 heterojunction catalyst in CO_2_ photoreduction was proposed based on the above results (Fig. 3g and h). After recombination, free electrons in BP with higher Fermi energy levels migrate spontaneously to SNO until they reach a Fermi energy balance. In this process, due to the absence of electrons, there is a positive charge near BP, and the energy band bends upward, while the energy band of SNO bends downward. Therefore, an internal electric field (IEF) is generated between SNO and BP, which is consistent with the following DFT calculation results. Driven by the IEF, the photogenerated electrons in the SNO CB are transferred to the BP CB to promote charge separation and improve the photocatalytic efficiency. Because of the bending of the energy band, the holes in the SNO VB are not conducive to transfer to the VB of BP, so the oxidation of H_2_O to O_2_ occurs in the SNO VB. In addition, different Er levels have different effects on the improvement of photocatalytic performance (Fig. 3h).

### DFT calculations and discussion

**Fig. 4:**
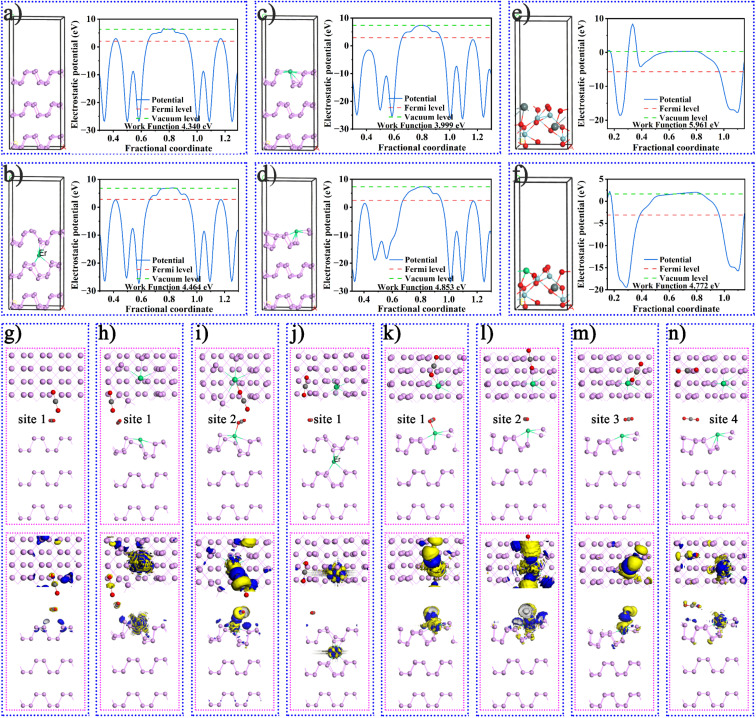
The optimized geometric structures and the calculated work functions for the (001) surface of (a) BP, (b–d) BPEr where Er occupies different P positions, (e) SNO, and (f) SNOEr. The CO_2_ adsorption sites and the corresponding charge density difference of (g) BP and (h–n) BPEr in which Er replaces different P positions.

As mentioned above, more in-depth research and theoretical calculations are needed to verify the mechanism of improving the catalytic activity of RE ions. Fig. S23† shows the optimized geometric structures of BP, BPEr, SNO, and SNOEr. The results of the band structures and density of states in Fig. S24† indicated that the band gap values were 0.510, 0.411, 2.111, and 2.262 eV for BP, BPEr, SNO, and SNOEr, respectively. The f-orbitals of Er mainly affect the valence band of both BP and SNO. The results of the optimized geometric structures of BP and BPEr in Fig. 4 indicate that the Er single atoms on the BP surface will cause surface reconstruction, thus improving the CO_2_ adsorption capacity of BP, which will be proved later. The calculated work functions are separately 4.340, 4.464, 3.999, and 4.853 eV for the (001) surface of BP and BPEr where Er occupies different P positions. Obviously, the work function decreases after surface reconstruction, and it is easier for the electrons in BP to escape and participate in the surface chemical reaction. Similarly, the work function of SNO decreases after Er doping. In addition, the Fermi energy level of SNO is lower than that of BP, and electrons at the interface are transferred from BP to the SNO surface, thus forming an internal electric field between BP and SNO, which will be discussed in detail later.

The configurations of CO_2_ adsorbed BP, BPEr, SNO, and SNOEr are shown in Fig. 4g–n and S25.† The CO_2_ adsorption energy of BP is −0.03 eV (Table S4†). While for BPEr (configuration I), the CO_2_ adsorption energies are −0.88 and −0.18 eV for different adsorption positions. For BPEr (configuration II), the CO_2_ adsorption energy is only −0.02 eV. For BPEr (configuration III), the CO_2_ adsorption energies are −0.46, −0.21, and −0.08 eV for different adsorption positions. The CO_2_ adsorption energies of SNO and SNOEr are −0.28 and −2.38 eV, respectively. The Mulliken charge of O and C of CO_2_ adsorbed on the BP surface is −0.49, −0.48, and 0.97 *e*, respectively. For site 1 and site 3 of BPEr(iii) in Table S5,† the sum of Mulliken charges of O and C of adsorbed CO_2_ is negative.

**Fig. 5:**
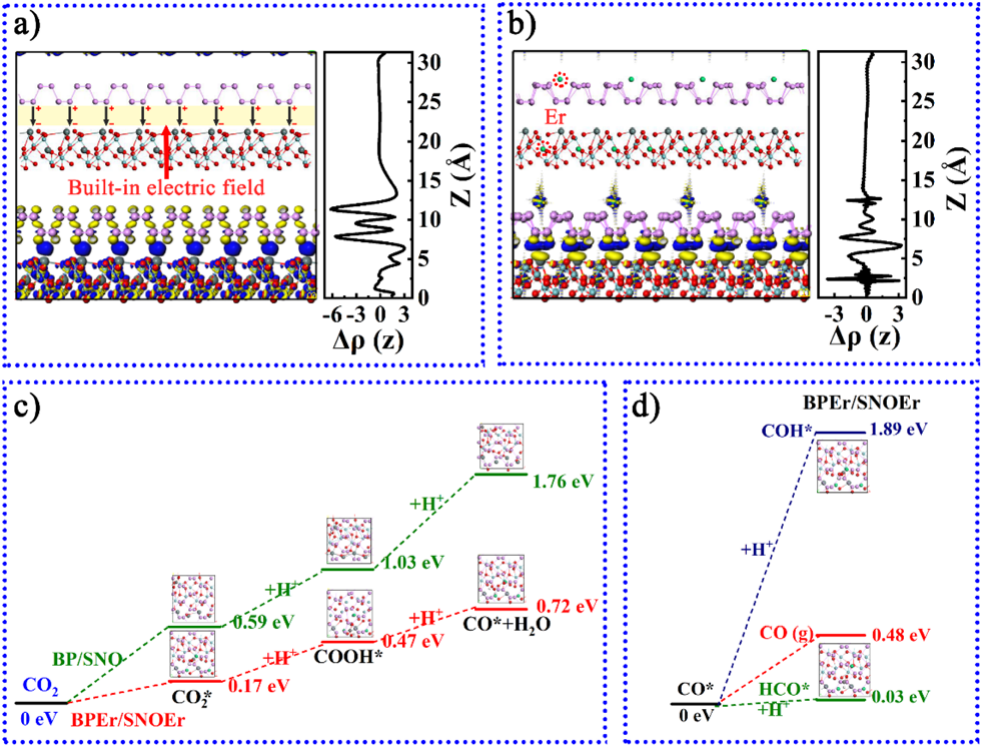
Optimized geometric structures and charge density difference of (a) BP/SNO and (b) BPEr/SNOEr heterojunctions. Calculated trends in the Gibbs free energy change of (c) the rate determination steps and (d) the selectivity determination steps.

As mentioned above, an internal electric field can form at the interface between BP and SNO (Fig. 5a). Fig. 5b shows the charge density difference of the heterojunction after the introduction of Er single atoms. The blue and yellow areas represent the increase and decrease in electron density, respectively. During the photocatalytic reaction, the charge can easily be transferred from the SNO CB to the BP CB driven by the built-in electric field.

To further study the influence of Er single atoms on the photocatalytic process, the Gibbs free energy change of the key carboxyl intermediate COOH* over BP/SNO and BPEr/SNOEr was calculated, as shown in Fig. 5c. Here, the COOH* is the key carboxyl intermediate in the rate measurement step CO_2_* + H^+^ + e^−^ → COOH* in the photoreduction process of CO_2_* to CO*. The CO_2_ adsorption values of BP/SNO and BPEr/SNOEr are 0.17 and 0.59 eV, showing that Er single atoms can promote the CO_2_ adsorption ability. The CO_2_* → COOH* energy changes on BP/SNO and BPEr/SNOEr are 0.44 and 0.30 eV, respectively. The energy change of COOH* → CO* + H_2_O over BPEr/SNOEr is also lower than that over BP/SNO. To explore the selectivity of BP/SNO and BPEr/SNOEr, the energy changes of CO* → CO, CO* → COH*, and CO* → HCO* were studied (Fig. 5d). The minimum energy change required for CO* → HCO* over BPEr/SNOEr is 0.03 eV, which is lower than that for CO* → CO. In general, it is much easier to generate CO than CH_4_ during CO_2_ photoreduction. Compared with the reported results in the literature, the generated CH_4_ in this work is relatively high, which is consistent with the calculated results above.^18^

## Conclusions

It is expected that efficient CO_2_ photoreduction may be realized by using the synergism of RE single atoms and heterojunctions. Here, excellent photocatalytic performance is obtained by constructing a BPEr/SNOEr heterojunction as scheduled, which was studied by combining steady-state, transient, and ultrafast spectral analysis techniques with quasi *in situ* XPS measurements and DFT theoretical calculations. The results indicated that the combination of BP not only broadens the absorption range of the catalyst, but also generates O vacancies on the surface of SNO, which is conducive to the adsorption of CO_2_ on the catalyst, thereby improving the photocatalytic activity. Most importantly, the Er single atom anchored heterojunction showed the highest photocatalytic activity after sample optimization. The CO yield (19.01 μmol h^−1^ g^−1^) of BPEr/SNOEr-10 is 3.4 and 10.7 times that of pure SNO and BP, and the CH_4_ yield (8.89 μmol h^−1^ g^−1^) is 1.5 times and 10.1 times that of pure SNO and BP, respectively. According to the J–O theory of lanthanide ions, the probability of radiative and non-radiative transitions between different energy levels is different due to the different electronic layer structures of the different rare earth ions, resulting in different rare earth ions having different effects on photocatalysis. Combined with the above rules and the CO_2_ photoreduction test under a single wavelength excitation, it is verified that the ^4^F_7/2_ → ^4^I_15/2_ and ^2^H_11/2_ → ^4^I_15/2_ emissions of Er in BPEr/SNOEr can be more easily absorbed by SNO and BP, which improves the utilization of light and thus improves the photocatalytic efficiency. The results of DFT calculations and photocatalytic characterization under single wavelength light excitation show that the Er single atoms not only can cause surface reconstruction, adjust the active site of BP, and promote light absorption with the help of 4f levels, but also can reduce the energy change value in the key steps (CO_2_* + H^+^ + e^−^ → COOH* and COOH* → CO* + H_2_O) of the photocatalytic CO_2_ reduction process. Based on this, a possible photocatalytic mechanism was proposed, which was expected to provide a unique insight into the design and mechanism exploration of efficient photocatalysts based on lanthanide single atoms.

## Data availability

The source data is available from the corresponding author upon reasonable request.

## Author contributions

H. F. and G. W. designed and directed this work, carried out theoretical research, and revised the manuscript. M. L. completed major experiments, analyzed and processed data, and wrote the manuscript. J. W. helped carry out photocatalytic and electrochemical tests. Q. W. and H. L. helped complete the sample synthesis experiment. All the authors discussed the results and commented on the manuscript.

## Conflicts of interest

There are no conflicts to declare.

## Supplementary Material

SC-015-D3SC05464K-s001
