# Supplementary material for: Study on synergistic effects of 4f levels of erbium and black phosphorus/SnNb2O6 heterostructure catalysts by multiple spectroscopic analysis techniques
Source: Chem Sci. 2024 Jan 5;15(5):1860–9. doi: 10.1039/d3sc05464k (PMC10829003; doi:10.1039/d3sc05464k)
Supplement: SC-015-D3SC05464K-s001 [file SC-015-D3SC05464K-s001.pdf]

Electronic Supplementary Material (ESI) for Chemical Science.
This journal is © The Royal Society of Chemistry 2023

Supporting Information

Study on Synergistic Effects of 4f Levels of Erbium and Black Phosphorus/$SnNb_2O_6$ Heterostructure Catalysts by Multiple Spectroscopic Analysis Techniques

Minze Li, Jingzhen Wang, Qiuye Wang, Honglai Lu, Guofeng Wang, and Honggang Fu**

Method

Chemicals and Materials: All reagents and solvents, including analytical-grade niobium pentaoxide (Nb_2O_5), potassium hydroxide (KOH), tin dichloride dihydrate ($SnCl_2{\cdot}2H_2O$), hydrochloric acid (HCl), $Er(NO_3)_3{\cdot}6H_2O$, red phosphorus, tin powder, iodine, N-Methyl pyrrolidone (NMP) and ethanol were used as received without further purification.

Synthesis of $SnNb_2O_6$ (SNO) and $SnNb_2O_6$:Ln^{3+} (SNOLn, Ln = Ce, Eu, Gd, Dy, and Er): For the synthesis of the SNO nanosheets, 0.5 g of Nb_2O_5 and 2.24 g of KOH were dissolved in 35 mL of deionized water. After stirring for 30 minutes, the above solution was transferred to a 50 mL Tefion-lined stainless-steel autoclave and heated at 180 °C for 48 h. After cooling to room temperature, the pH value of the solution was further adjusted to about 7 by HCl under stirring, and then the white $Nb_2O_5{\cdot}nH_2O$ suspension was obtained. 0.4244 g of $SnCl_2{\cdot}2H_2O$ was slowly added to the above white $Nb_2O_5{\cdot}nH_2O$ suspension under vigorous stirring. After 30 minutes, the solution was further transferred to a Teflon-lined stainless-steel autoclave and kept at 180 °C for another 48 h in the oven. After cooling to room temperature, the precipitate at the bottom was centrifuged, washed respectively by deionized water and ethanol for 3 times, dried at 60 °C in an oven, and finally the SNO nanosheets were obtained successfully. The synthesis process of SNOLn is similar to that of SNO, except that the 0.5 mol% $SnCl_2{\cdot}2H_2O$ is replaced by $Ln(NO_3)_3{\cdot}6H_2O$ in the initial reaction process.

Synthesis of black phosphorus (BP) and Er single atom anchored BP (BPEr): The phase transformation reaction with red phosphorus (RP) as a raw reactant is a common method to prepare bulk BP materials. First, red phosphorus (450 mg), tin powder (35 mg), and iodine (20 mg) were thoroughly ground and mixed. The mixture was placed into a quartz tube in a glove box, and then the quartz tube was filled with argon and finally pre-encapsulated. The pre-encapsulated quartz tube was re-encapsulated in a 15 cm long tube using a hydrogen-oxygen flame machine. The encapsulated long glass tube was put into a muffle furnace, heated according to the preset procedure, cooled to room temperature, and opened. The sample was labeled as bulk BP. To ground bulk BP

into a fine powder, the bulk BP was placed in a ball mill, and NMP solution was added at a solid-to-liquid ratio of 100:1 (mg/mL), followed by an appropriate amount of stainless steel balls, covered with a lid and placed in a ball mill. The speed and running time of the planetary ball mill were set at 400 r/min and 4 hours, respectively. After ball milling, the remaining unexfoliated particles are removed by settling for one hour. BP nano sheet dispersion was collected and washed with ethanol for 6 times to remove residual NMP by high-speed centrifugation. The sample was labelled as BP. The BPEr was synthesized by a facile ultrasonic method. 0.304 g of the milled BP and 250 μL of $Er(NO_3)_3 \cdot 6H_2O$ (0.2 mol/L) were dispersed to 20 mL of NMP, and then the mixture solution was treated with sonication for 4 h in the ice-water bath. Finally, the BPEr was collected by the same way as BP.

Synthesis of BPEr/SNOEr: A certain amount of BPEr and SNOEr were added to 20 mL of NMP, and then the mixture solution was treated with sonication for 4 h in the ice-water bath. Finally, the BPEr/SNOEr was collected by the same way as BP and BPEr. The synthesis parameters and sample abbreviations are shown in Table S2.

Characterization: X-ray diffraction (XRD) patterns were recorded on a Bruker D8 Advance diffractometer equipped with Cu Kα radiation (λ = 1.5406 Å, 40 kV, 40 mA). The scanning electron microscope (SEM, Hitachi, S-4800) and transmission electron microscope (TEM, JEOL, JEM-2100) were used to characterize the morphology and size of the samples. The Fourier transform infrared (FT-IR) spectra were recorded with KBr pellets in the range of 4000-400 cm^{-1} on a Perkin-Elmer Spectrum One spectrometer. UV-vis diffuse reflectance spectra (UV-vis DRS) were recorded with a spectrophotometer (SHIMADZU UV-2550). The photoluminescence (PL) spectra were measured at room temperature by using a Hitachi F-4600 fluorescence spectrophotometer equipped with a 150 W Xe lamp. The elemental composition of the catalyst was measured by using X-ray photoelectron spectroscopy (Kratos-AXIS ULTRA DLD device with Al (single) X-ray source).

Photoelectrochemical measurement: A computer-controlled CHI-660 electrochemical workstation (Chenhua Instrument, Shanghai, China), with a 300 W Xe

lamp as the light source, was used to characterize the photoelectrochemical performance (PEC). A three-electrode configuration with the sample membrane as the working electrode, saturated Ag/AgCl as the reference electrode, platinum foil as the counter-level, and 0.5 M Na_2SO_4 aqueous solution as the electrolyte was used for photocurrent measurement. 0.1 g of the photocatalyst was dispersed into 1 mL of isopropanol, 0.05 g of Macrogol-2000 was added under continuous stirring, and the mixture was mixed with ultrasonic for 10 minutes, and then the suspension was stirred vigorously for 30 minutes. Then, 0.05 mL of acetylacetone was added, and the obtained solution was kept under sufficient stirring for one week to obtain a desired paste. 1cm × 1cm conductive fluorine doped tin oxide (FTO) coated glass was used as the substrate. The prepared paste was coated on the substrate by a scraper method. Finally, the electrode was dried at room temperature and annealed at 150 °C for 2 hours in a N_2 atmosphere. Before the experiment, high-purity nitrogen needs to be introduced into the electrolyte for 30 minutes, and then the test is carried out. The calculation formula of the guide belt is as follows: $E_{(NHE)} = E_{(Ag/AgCl)} + 0.197$ (pH = 7). Disperse 25mg of catalyst powder in 1 mL of ethanol, and then drop the dispersed liquid in 1cm × 1cm carbon paper is used as the working electrode. Linear sweep voltammetry (LSV) measurement was carried out in 0.5 M KOH solution saturated with CO_2 and N_2, and maintain gas flow throughout the test.

EPR measurement: 50 μL of catalyst suspension with a concentration of 4 g/L was injected into 500 μL of specific capture agent solution with a concentration of 50 mmol/L (·OH was measured by DMPO aqueous solution). After mixing evenly, the mixture was transferred into the capillary column, sealed with vacuum grease, and then transferred into the quartz sample tube, placed in the resonator of electron spin resonance spectrometer, and tested after a certain time of light reaction.

Photocatalytic measurement: A certain amount of powder sample was dispersed into a cylindrical steel reactor with a volume of 100 mL and an area of 3.5 cm^2, and 4 mL of water was added. After passing through water, the high-purity CO_2 gas enters the reaction device to reach the ambient pressure, further remove the original air and

impurities in the reaction device, and achieve the adsorption and desorption balance before irradiation. A 300 W Xe lamp (PLSSXE300/300UV, Perfect light, Beijing) with a 420 nm cut-off filter was used as the light source for the photocatalytic reaction, and the sample was continuously irradiated with a light intensity of 455 mW cm^{-1} for 6 hours. Quantitative chromatograph (GC2002) was used to analyze and detect the produced gas. In order to ensure the accuracy of the data, all reported photocatalytic reactions have been tested more than five times. For the stability experiment, the photocatalytic reaction was carried out for 5 cycles, and the time of each photocatalytic reaction was 6 hours.

It is noted that the H_2 was not detected in the gas products. We conducted chemical reaction equilibrium calculations on the product based on the following reaction equation,

$$2H_2O(l)+4h^+ \rightarrow O_2(g)+4H^+ \quad (1)$$

$$CO_2(g)+8e^-+8H^+ \rightarrow CH_4(g)+2H_2O(l) \quad (2)$$

$$CO_2(g)+2e^-+2H^+ \rightarrow CO(g)+H_2O(l) \quad (3)$$

it can be concluded that the oxidation reaction is the oxidation of H_2O by holes to O_2 and H^+, and H^+ will participate in the reaction and produce CH_4 and CO. In these process, taking BPEr/SNOEr-10 as an example, the experimental yield of O_2 is 27.05 μmol h^{-1} g^{-1}, and based on the stoichiometric ratio, the yield of H^+can be obtained as 108.20 μmol h^{-1} g^{-1}. These H^+ were not completely consumed to produce CH_4 during the 8-electron process, and some of them participated in the reaction to produce CO during the 2-electron process. Due to the experimental yields of 8.89 and 19.01 μmol h^{-1} g^{-1} for CH_4 and CO, the total amount of H^+ involved in the 8-electron process and 2-electron process can be obtained from the stoichiometric ratio in the above reaction formula as follows:

$$\nu(H^+) = 8\nu(CH_4) + 2\nu(CO) = 8 \times 8.89 + 2 \times 19.01 = 109.14 \ (\mu mol\ h^{-1}\ g^{-1})$$

Where $\nu(H^+)$, $\nu(CO)$ and $\nu(CH_4)$ represent the yield of products, respectively.

Obviously, the amount of H^+ involved in the reaction is greater than that produced during the oxidation process, so there is no excess H^+ reduced to H_2. In fact, we also did not detect H_2 in the experiment.

The selectivity of CO and CH_4 is calculated according to the following formula:

CO selectivity = $2\nu(CO)/[2\nu(CO)+8\nu(CH_4)] \times 100\%$

CH_4 selectivity = $8\nu(CH_4)/[2\nu(CO)+8\nu(CH_4)] \times 100\%$

For the calculation of the efficiency ratios of samples containing Er and samples without Er under different single wavelength light is based on the method reported in the literatures (*ACS Nano,* 2014, *8*, 7229; *JMCA*, 2022, *10*, 5990). First, the calculation of quantum efficiency (QE) for CO and CH_4 generations were calculated using the following equation: QE = (2×CO generation rate + 8×CH_4 generation rate)/(absorption rate of incident photon). And then, the Efficiency ratio = (QE of sample containing Er)/(QE of sample without Er) = (2×CO generation rate + 8×CH_4 generation rate of sample containing Er)/(2×CO generation rate + 8×CH_4 generation rate of sample without Er). The photocatalytic efficiency under single wavelength is normalized based on the light intensity of the Xe lamp.

The impact of radiative transitions on electron transfer should be viewed from two aspects: one is the impact of the energy generated by fluorescence on charge transfer, and the other is the influence of radiative transitions on the electron population of highly excited states in Er, which may affect electron transfer.

(1) As mentioned by the reviewer, non-radiatively relaxing electrons are considered to be the most direct energy for photocatalytic surface reactions. But for radiative transitions, the photoluminescence intensity usually mainly reflect the degree of recombination of photogenerated charge carriers, and the significant PL intensity is mainly due to the high recombination of photogenerated carriers, which is not conducive to photocatalytic reactions. On the other hand, the energy of photoluminescence is mainly radiated in the form of light energy, so it has little impact on the transmission of electrons. The results of photoluminescence (Figure S19 and 20) indicate that the lower the intensity of photoluminescence, the better the photocatalytic performance, which is consistent with our expected results.

(2) From the energy level diagram of Er^{3+} ions (Figure 3h), it can be seen that the photoluminescence process can reduce the electron population on highly excited states and increase the electron population on the ground state. The electron population on

highly excited states will affect the probability of non radiative transitions, thus indirectly affecting electron transfer. For example, the emissions of $^2H_{9/2}\rightarrow^4I_{15/2}$, $^4F_{3/2}(^4F_{5/2})\rightarrow^4I_{15/2}$, $^4F_{7/2}\rightarrow^4I_{15/2}$, $^2H_{11/2}\rightarrow^4I_{15/2}$, $^4S_{3/2}\rightarrow^4I_{15/2}$, $^4F_{9/2}\rightarrow^4I_{15/2}$ directly affect the electronic populations of $^2H_{9/2}/^4F_{3/2}(^4F_{5/2})/^4F_{7/2}/^2H_{11/2}/^4S_{3/2}/^4F_{9/2}$, and a decrease in electronic population will reduce the probability of non radiative transitions, thereby affecting electron transfer.

Femtosecond transient absorption measurements: Femtosecond transient absorption (fs-TA) measurements were performed on a Helios (Ultrafast systems) spectrometers using a regeneratively amplified femtosecond Ti:sapphire laser system (Spitfire Pro-F1KXP, Spectra-Physics; frequency, 1 kHz; max pulse energy, ~8 mJ; pulse width, 120 fs) at room temperature. Finally, analyze the data through commercial software (Surface Xplorer, Ultrafast Systems).

Quasi in-situ XPS measurement: Quasi in-situ XPS measurement was performed at an ESCALAB 250 Xi electron spectrometer from ThermoFischer, America. The specific experimental process is as follows: firstly, measurements are conducted in a CO_2 filled atmosphere without visible light irradiation; Next, measure again after being exposed to visible light for 6 h in the same atmosphere.

Computational details for DFT calculations. The plane-wave ultrasoft (PWUS) pseudopotential method, as implemented in the Cambridge Sequential Total Energy Package (CASTEP) algorithm, was used to mimic all geometric optimizations, band structure, and the partial density of states (PDOS), work function, and charge density difference. The absorption spectra were obtained in CASTEP using the Perdew-Wang from 1991 (PW91) functional within the generalized gradient approximation (GGA). Furthermore, on-site Coulomb interactions are included for f orbital of Er (U = 6 eV) using the GGA+U method. The plane-wave expansion's cutoff energy was set to 700 eV. The Brillouin zone integration was performed with 3 × 4 × 1 k-points for geometry optimization. The criteria for convergence in the total energy, force, and displacement convergence threshold are 1.0×10^{-5} eV/atom, 0.05 eV/Å, and 0.005 Å, respectively. Geometry optimization, electronic structure, and optical property calculations were all

performed using spin-polarized magnetic computation. The Gibbs free energy change in photocatalytic CO_2 reduction is defined as ΔG = G (final state) – G (initial state) = $E_{reaction} + \Delta E_{zero} - T\Delta S$, where $E_{reaction}$ refers to the reaction energy, E_{zero} is zero vibration energy correction, ΔS represents the differences in entropy, and the reaction temperature is T.

Table S1. Control experiments to prepare $SnNb_2O_6$ and $SnNb_2O_6$:Ln^{3+}.

Samples	Names	Nb_2O_5 (mmol)	KOH (mmol)	$SnCl_2·2H_2O$ (mmol)	$Ln(NO_3)_3$ (mmol)	Gas yield (μmol/h/g)	
						CO	CH_4
$SnNb_2O_6$	SNO	1.88	40	1.88	0	5.60	5.95
$SnNb_2O_6$:Ce^{3+}	SNOCe	1.88	40	1.8706	0.0094	5.67	6.22
$SnNb_2O_6$:Eu^{3+}	SNOEu	1.88	40	1.8706	0.0094	6.04	4.99
$SnNb_2O_6$:Dy^{3+}	SNODy	1.88	40	1.8706	0.0094	6.50	5.98
$SnNb_2O_6$:Er^{3+}	SNO:0.25Er	1.88	40	1.8753	0.0047	6.71	5.87
	SNO:0.5Er	1.88	40	1.8706	0.0094	8.68	6.12
	SNO:0.75Er	1.88	40	1.8659	0.0141	6.63	5.77
	SNO:1.0Er	1.88	40	1.8612	0.0188	6.41	5.66
$SnNb_2O_6$:Gd^{3+}	SNOGd	1.88	40	1.8706	0.0094	3.49	5.57

Table S2. Control experiments to prepare BP, BPEr, BP/SNO, BP/SNOEr, and BPEr/SNOEr.

Samples	$SnNb_2O_6$ (g)	BP (g)	BPEr (g)	NMP (mL)	Gas yield (μmol/h/g)	
					CO	CH_4
BP	—	—	—	—	1.77	0.88
BPEr	—	—	—	—	2.10	0.96
BP/SNO-10	0.1	0.01	0	20	12.29	5.64
BP/SNOEr-1	0.1	0.1	0	20	13.34	7.17
BP/SNOEr-5	0.1	0.02	0	20	13.79	8.05
BP/SNOEr-10	0.1	0.01	0	20	16.95	8.61
BP/SNOEr-15	0.1	0.0067	0	20	16.34	7.52
BP/SNOEr-20	0.1	0.005	0	20	15.60	6.66
BPEr/SNOEr-10	0.1	0	0.01	20	19.01	8.89

Table S3. The carrier concentration obtained through Hall effect testing.

Samples	Carrier concentration (cm^{-3})
SNO	$7.05×10^{14}$
SNO:0.25Er	$1.09×10^{16}$
SNO:0.5Er	$3.14×10^{16}$

Table S4. The CO_2 adsorption energies of BP, BPEr, SNO, and SNOEr with different configurations and different adsorption positions. Here, different BPEr configurations correspond to the configurations in Figure 4.

Samples	Site 1	Site 2	Site 3	Site 4
BP	-0.03 eV	—	—	—
BPEr (I)	-0.88 eV	-0.18 eV	—	—
BPEr (II)	-0.02 eV	—	—	—
BPEr (III)	-0.46 eV	-0.46 eV	-0.21 eV	-0.08 eV
SNO	-0.28 eV	—	—	—
SNOEr	-2.38 eV	—	—	—

Table S5. Mulliken charges of O and C in CO_2 adsorbed on (001) surface for BP and BP:Er with different configurations. Here, different BPEr configurations correspond to the configurations in Figure 4.

Samples	Site 1	Site 2	Site 3	Site 4
BP	0.97, -0.49, -0.48	—	—	—
BPEr (I)	0.96, -0.48, -0.48	0.93, -0.49, -0.44	—	—
BPEr (II)	0.96, -0.48, -0.48	—	—	—
BPEr (III)	0.88, -0.47, -0.43	0.91, -0.45, -0.44	0.88, -0.48, -0.43	0.96, -0.48, -0.48

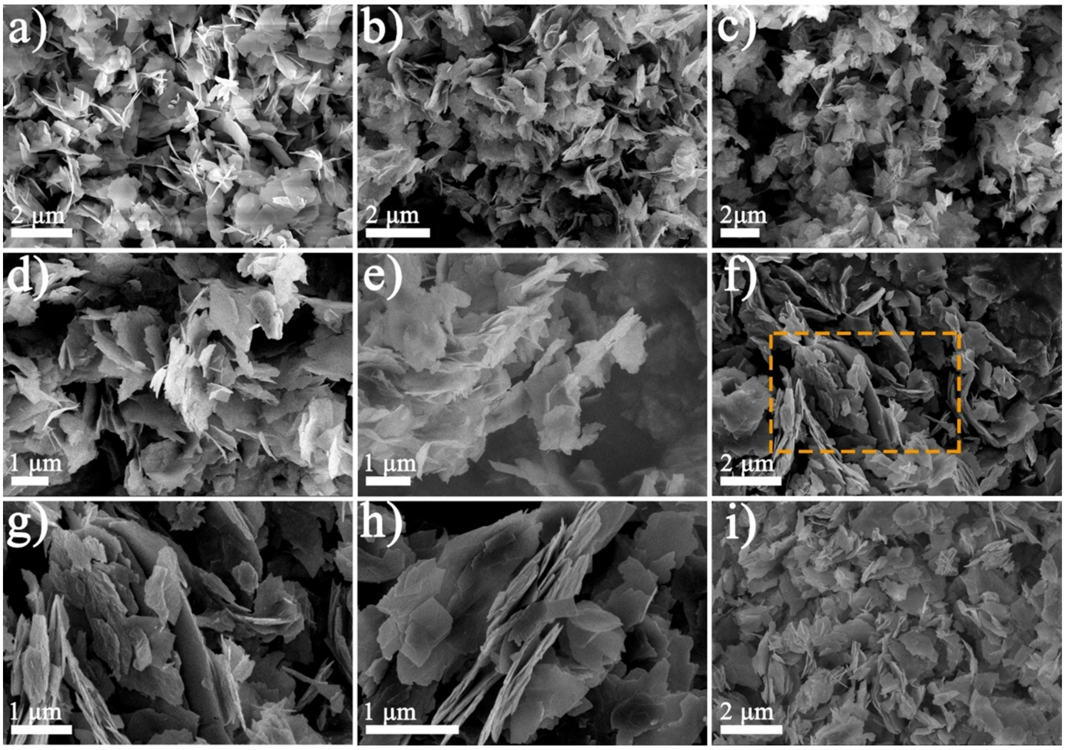

Figure S1. SEM images of (a) SNO, (b) SNOCe, (c) SNOEu, (d) SNODy, (e) SNOEr, (f,g) BP/SNOEr-5, (h) BP/SNOEr-10, and(i) BP/SNOEr-20.

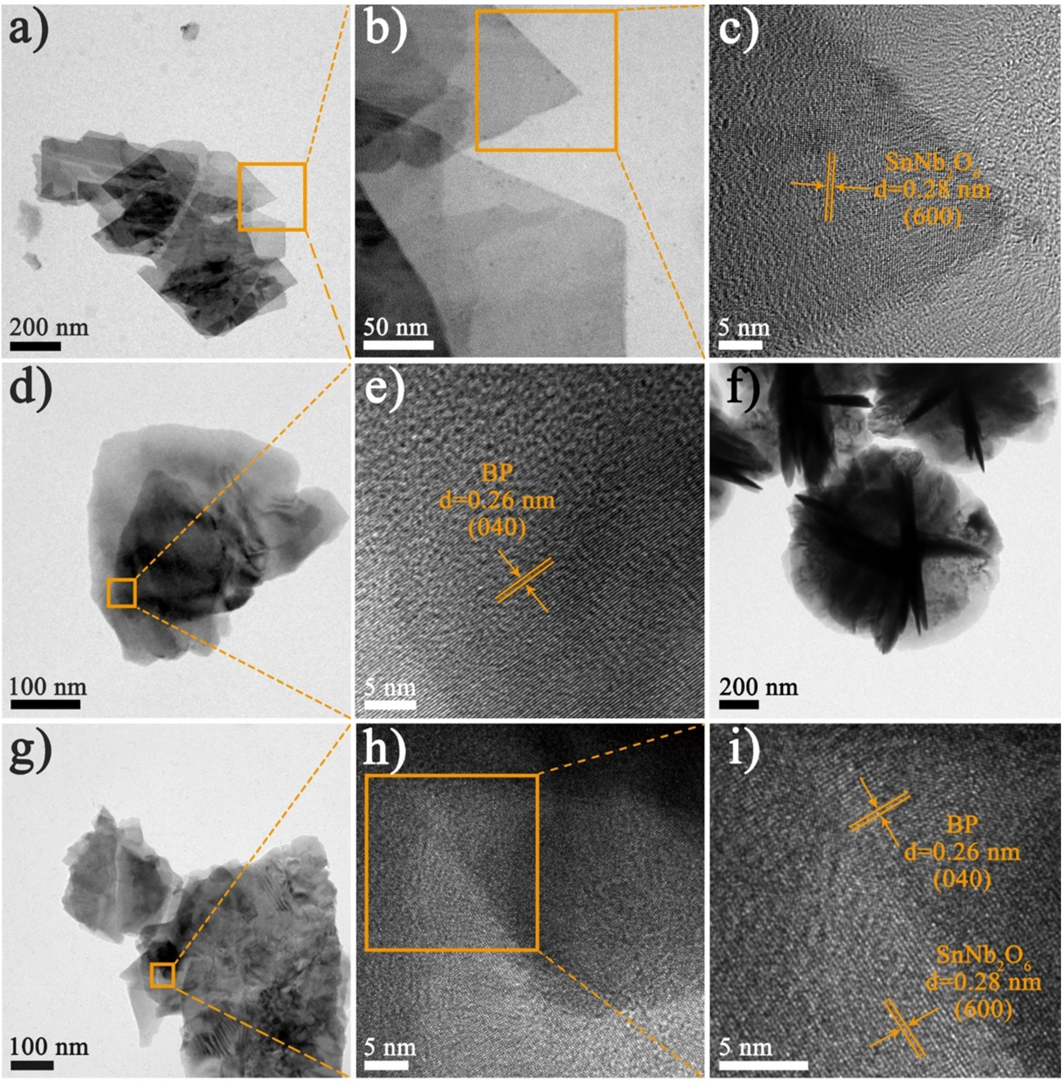

Figure S2. TEM and HRTEM images of (a-c) SNO, (d,e) BP, and (f-i) BPEr/SNOEr-10.

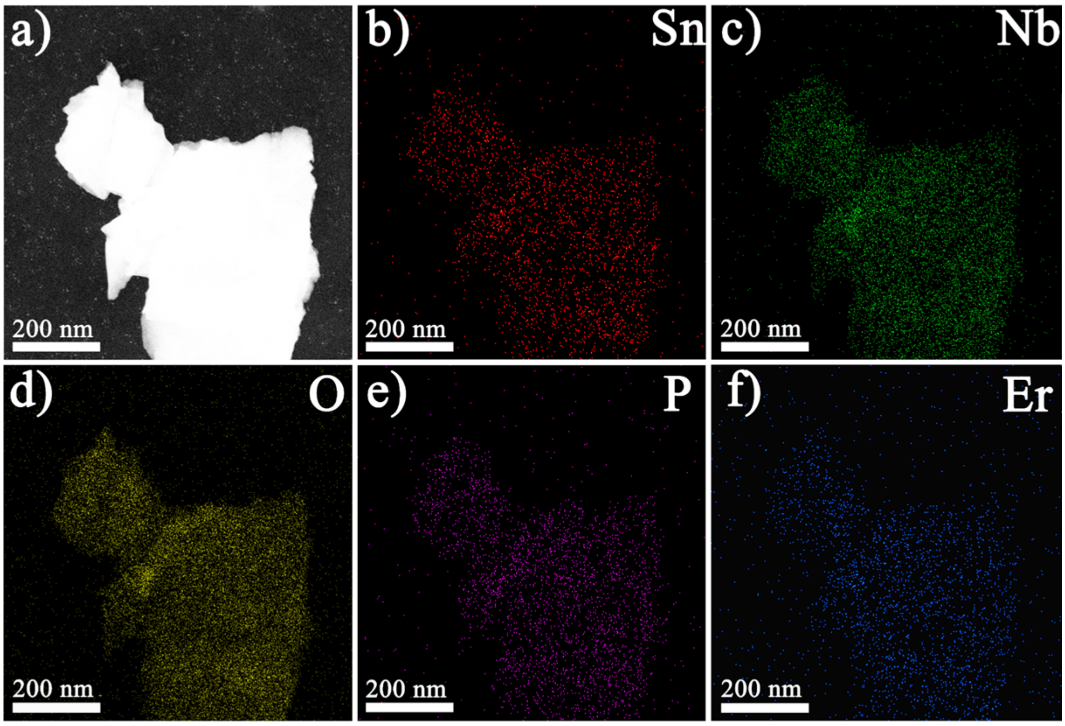

Figure S3. (a) STEM image and (b-f) EDX elemental mappings of BPEr/SNOEr-10.

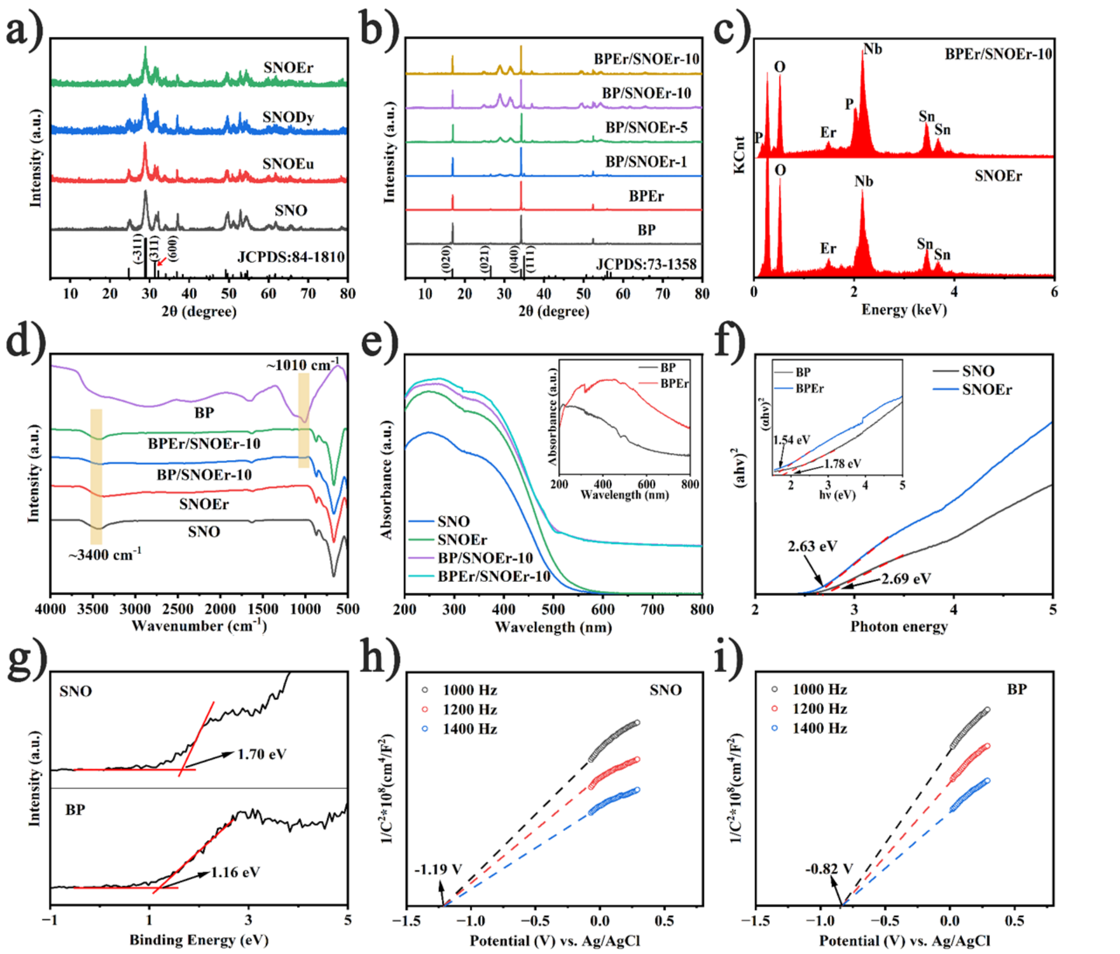

Figure S4. (a,b) XRD patterns, (c) EDS, (d) FT-IR spectra, (e) UV-Vis DRS spectra, (f) plots of $(ahv)^2$ versus photon energy, (g) XPS valence band spectra, and (h,i) Mott-Schottky plots of different samples.

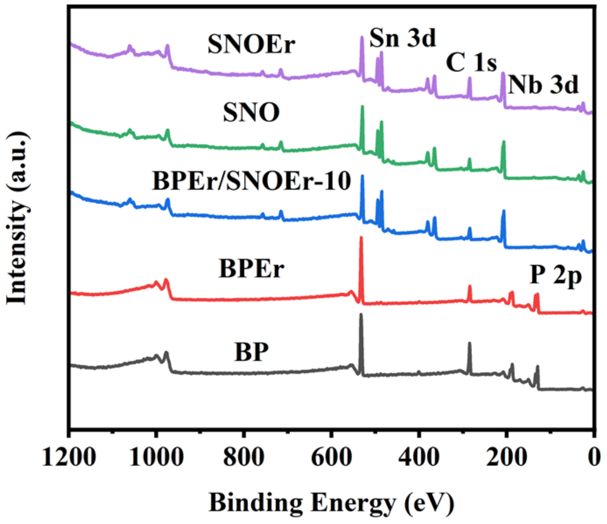

Figure S5. XPS survey spectra for BP, BPEr, SNO, SNOEr, and BPEr/SNOEr-10.

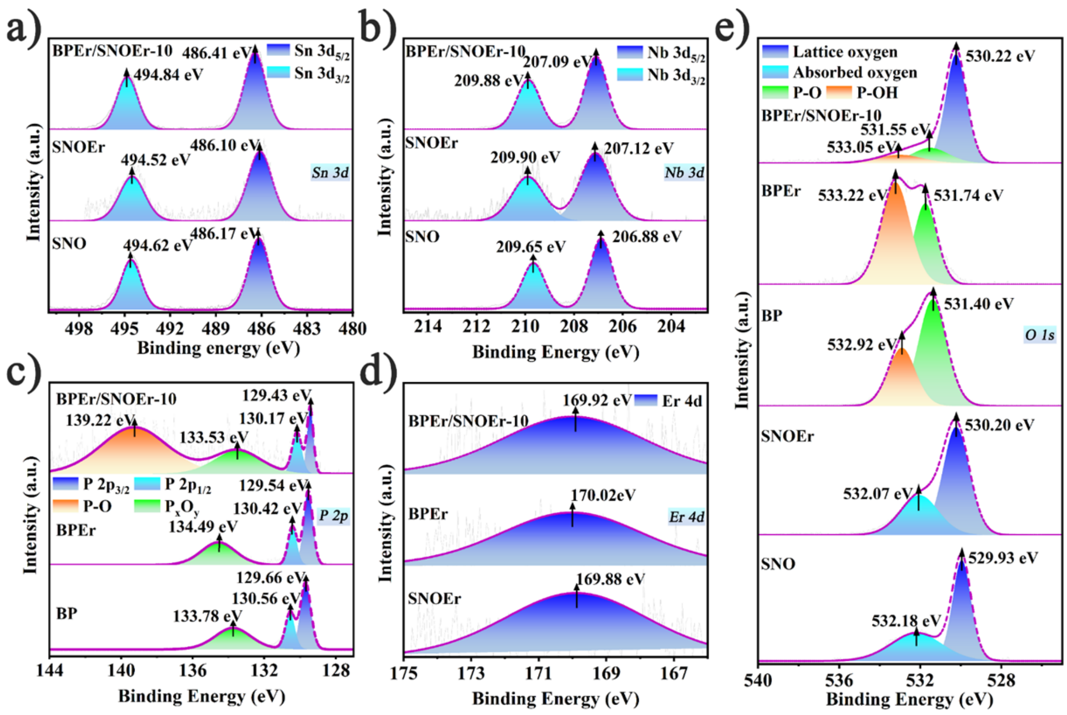

Figure S6. XPS spectra of different samples: (a) Sn 3d, (b) Nb 3d, (c) P 2p, (d) Er 4d, and (e) O 1s.

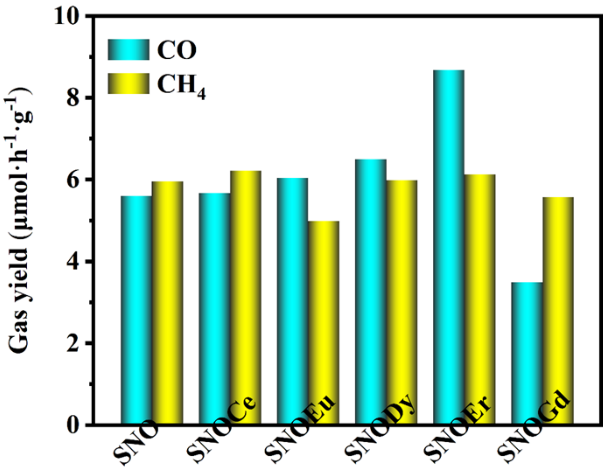

Figure S7. CO_2 photoreduction production rates of SNO and SNO:Ln (Ln = Ce, Eu, Dy, Er, and Gd).

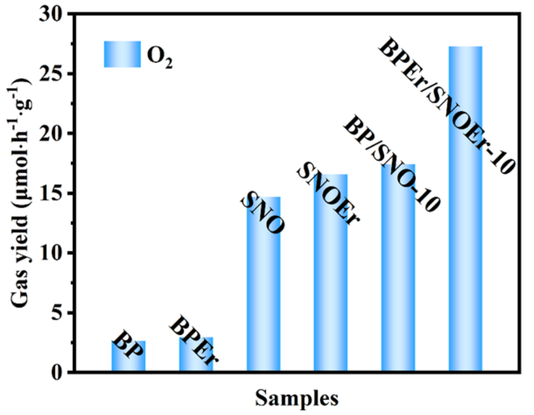

Figure S8. O_2 yield of catalyst under visible light irradiation.

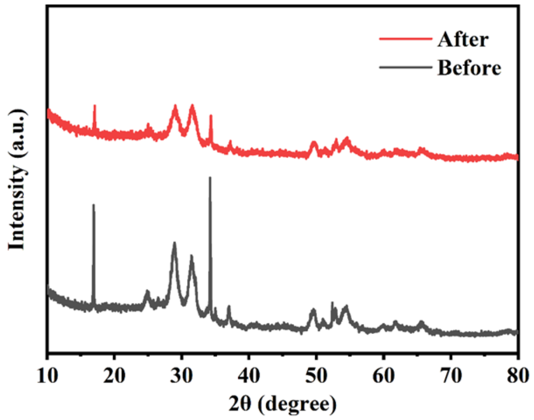

Figure S9. XRD patterns of BPEr/SNOEr-10 before and after photocatalysis test.

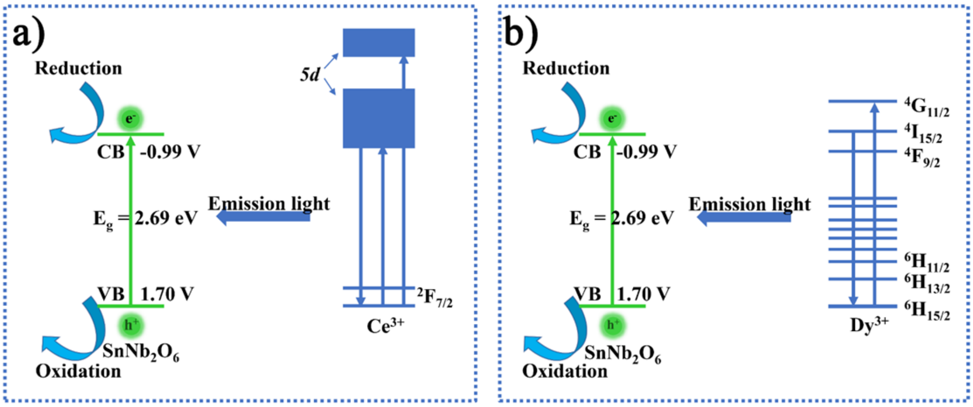

Figure S10. Schematic diagrams of visible light absorption process of (a) $SnNb_2O_6$:Ce and (b) $SnNb_2O_6$:Dy assisted by Ce and Dy ions. The emission light from 5d to 4f energy levels of Ce^{3+} can be further absorbed by $SnNb_2O_6$, so the photocatalytic performance of $SnNb_2O_6$:Ce can be improved. Similarly, the emission light between 4f energy levels of Dy^{3+} can also be further absorbed by $SnNb_2O_6$, so the photocatalytic performance of $SnNb_2O_6$:Dy can also be improved.

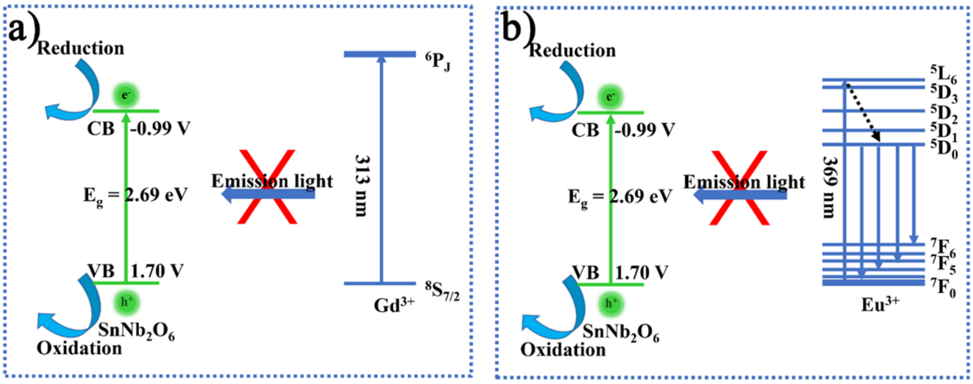

Figure S11. Schematic diagrams of the reason why Gd and Eu ions can't help $SnNb_2O_6$:Gd and $SnNb_2O_6$:Eu absorb visible light. For $SnNb_2O_6$:Gd, the $^6P_J \rightarrow ^8S_{7/2}$ emissions of Gd^{3+} is within the range of ultraviolet light and cannot absorb and emit visible light, so Gd^{3+} has no effect on the improvement of photocatalytic performance. For $SnNb_2O_6$:Eu, the non-radiative transition between $^5L_6 \rightarrow ^5D_0$ is usually very easy to occur, so most electrons in $^5L_6$ level are transferred to the $^5D_0$ level through the non-radiative transition, so the emissions of $^5D_0 \rightarrow ^7F_J$ is the main emissions. It is noted that the emission photon energy of Eu^{3+} is less than the photon energy required to excite $SnNb_2O_6$, so it cannot be absorbed by $SnNb_2O_6$ for the second time, so Eu^{3+} also has no effect on the improvement of photocatalytic performance.

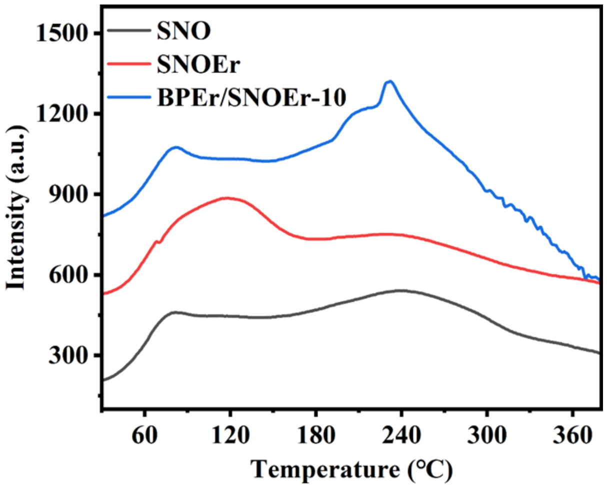

Figure S12. CO_2-TPD profiles of SNO, SNOEr, and BPEr/SNOEr-10.

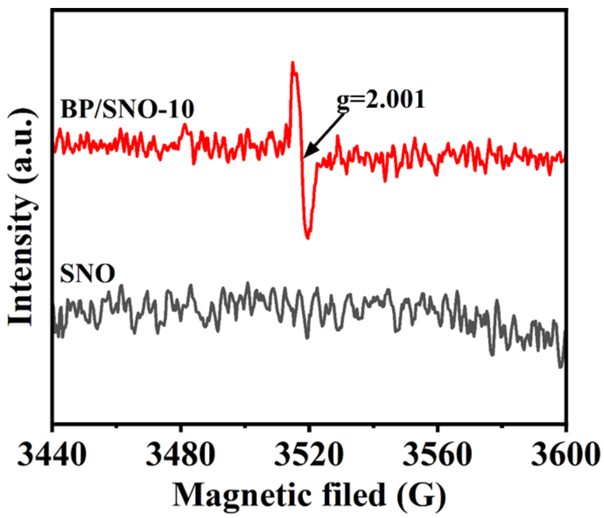

Figure S13. EPR spectra of SNO and BP/SNO-10 without additional light irradiation.

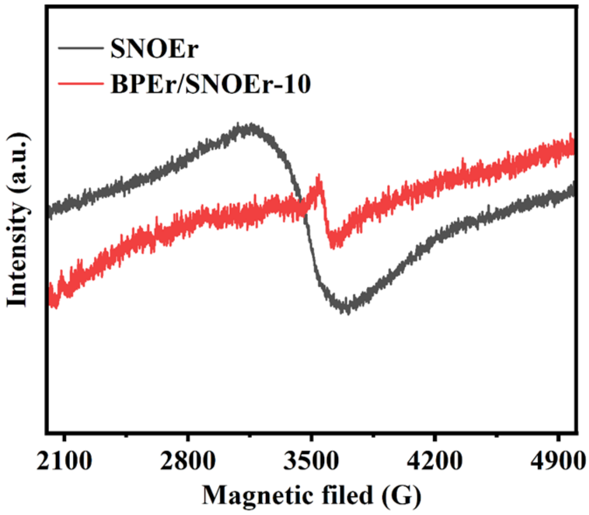

Figure S14. EPR spectra of SNOEr and BPEr/SNOEr-10.

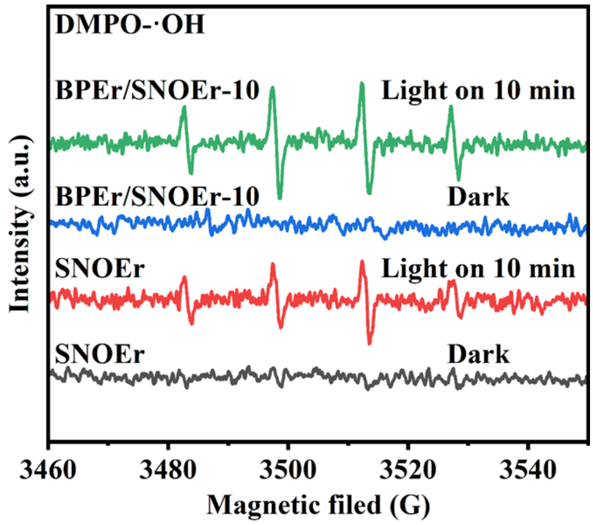

Figure S15. EPR spectra of SNOEr and BPEr/SNOEr-10 under dark and visible light irradiation in the presence of DMPO.

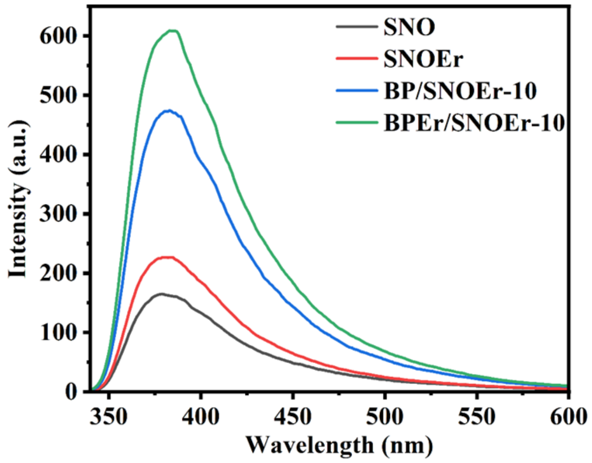

Figure S16. Spectral changes of hydroxyl radicals produced under 325 nm light excitation.

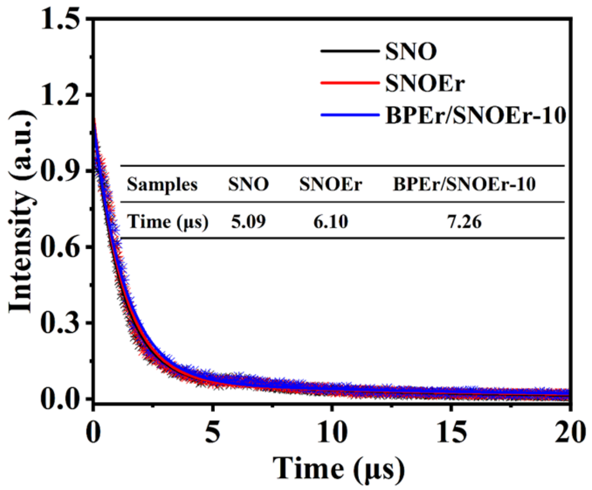

Figure S17. Luminescence decay curves (λ_{ex} = 325 nm).

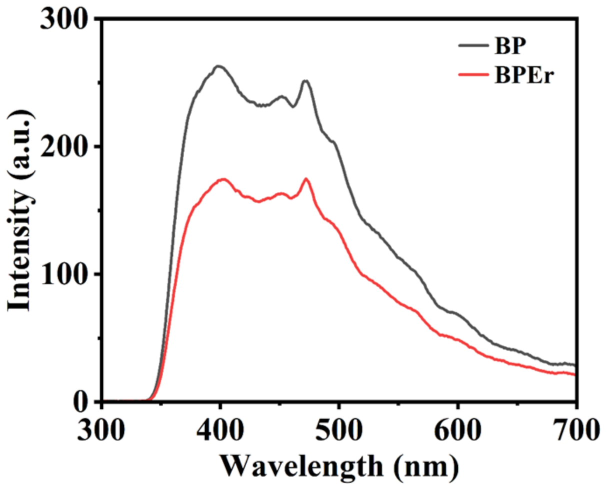

Figure S18. PL spectra (λ_{ex} = 325 nm) of BP and BPEr.

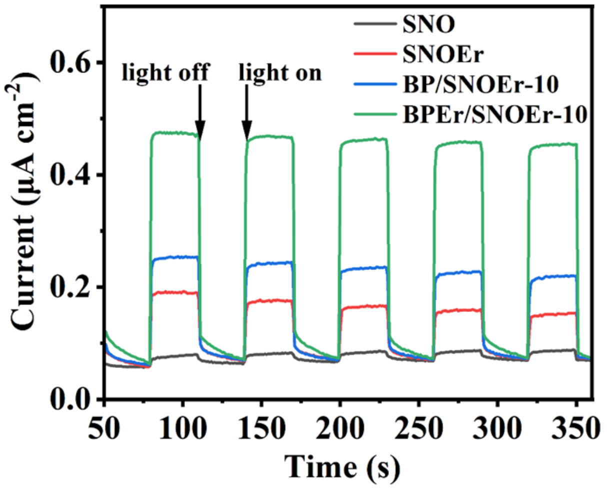

Figure S19. Transient photocurrent curves of different samples.

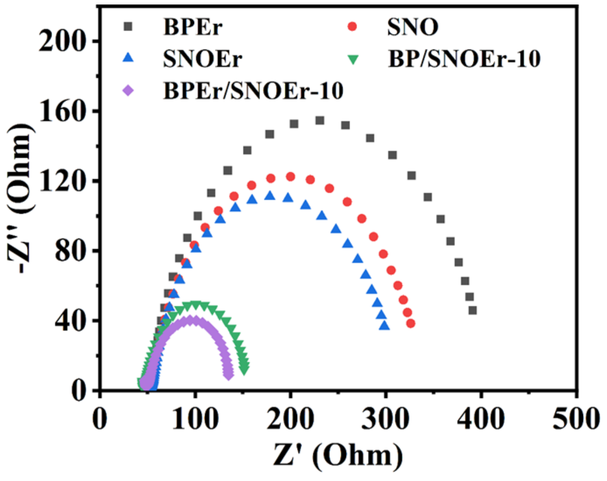

Figure S20. EIS spectra of different samples.

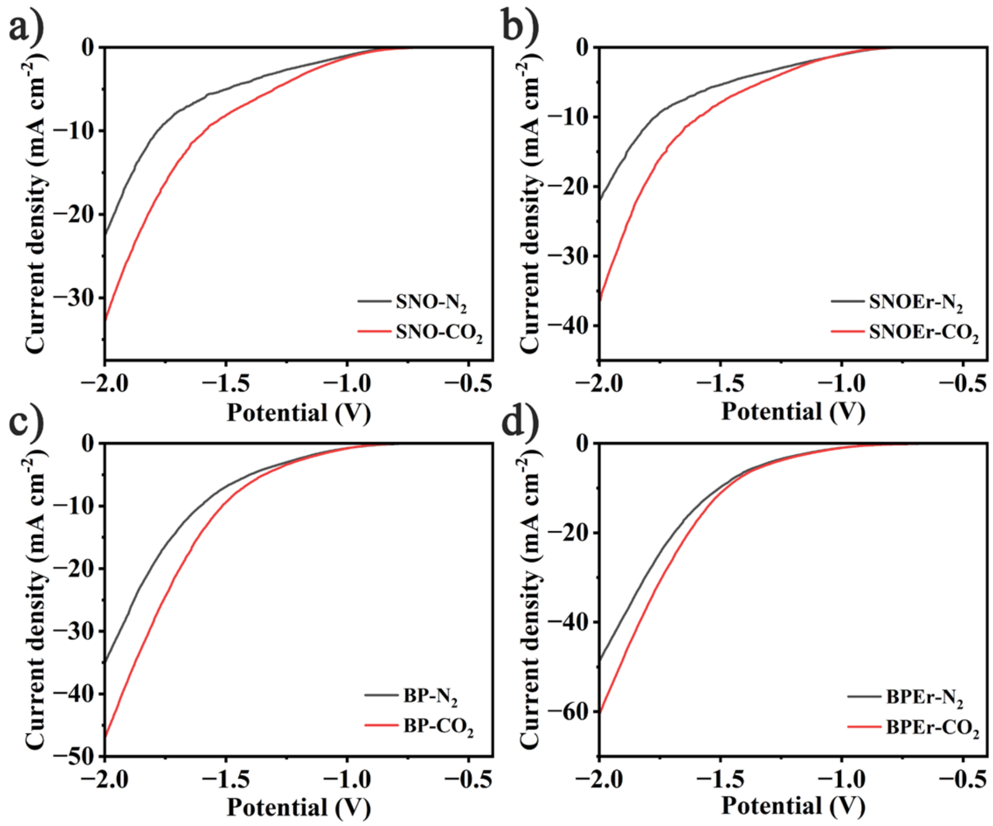

Figure S21. LSV curves of (a) SNO, (b) SNOEr, (c) BP and (d) BPEr in CO_2-saturated (red lines) and in N_2-saturated (black lines) $KHCO_3$ (0.5 M) solution.

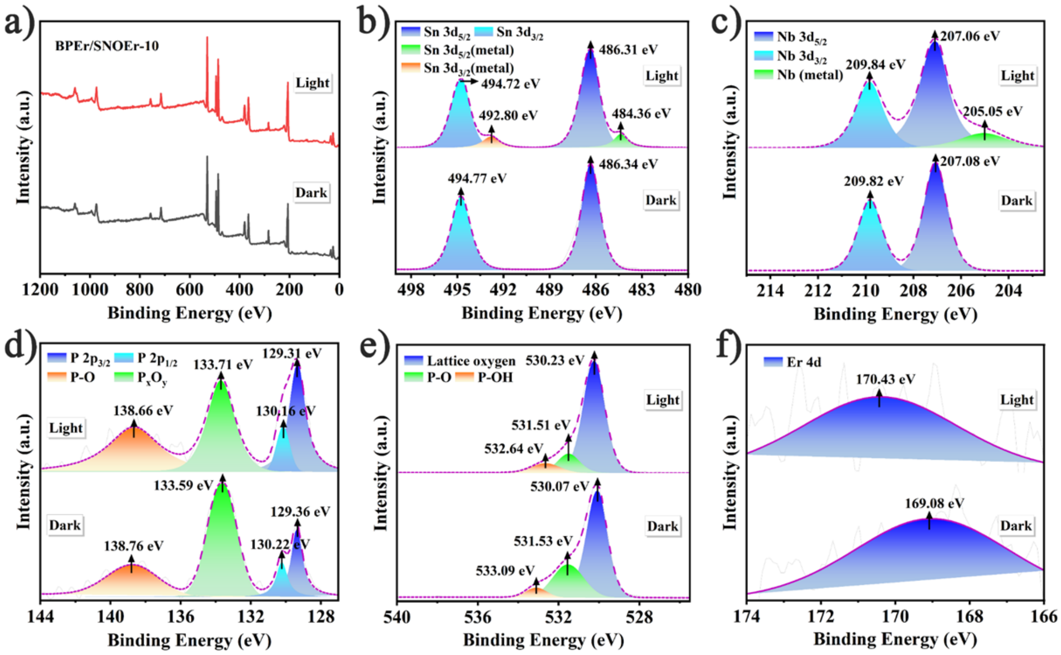

Figure S22. Quasi in-situ XPS spectra of different elements of BPEr/SNOEr-10 under and without visible light irradiation.

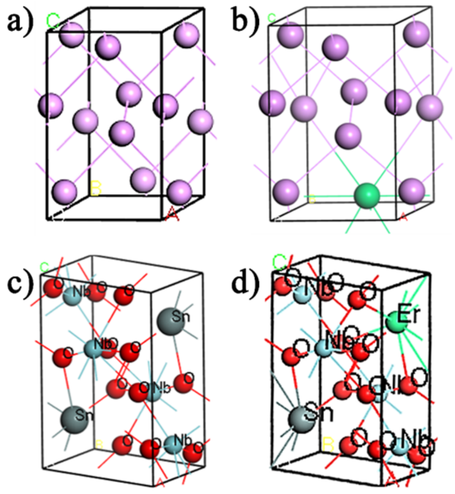

Figure S23. The geometric structures of (a) BP, (b) BPEr, (c) SNO, and (d) SNOEr.

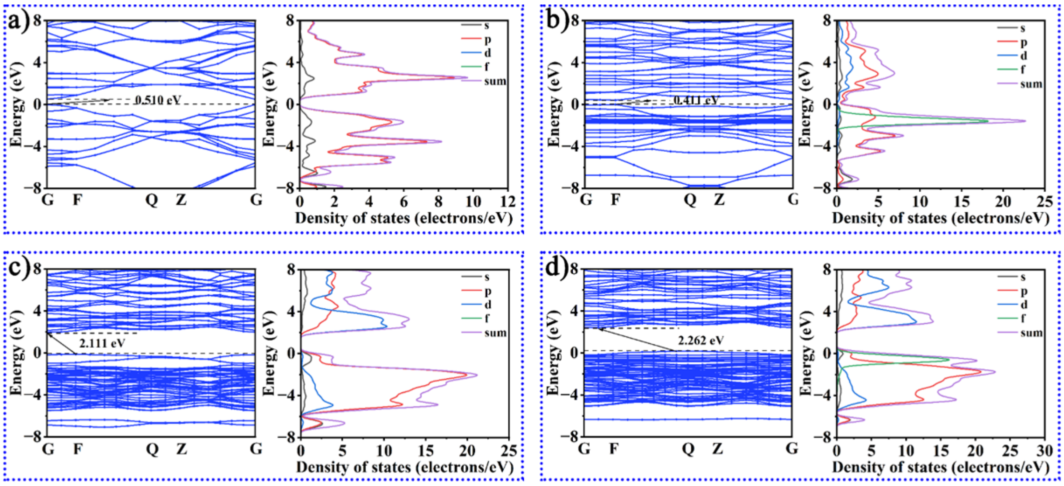

Figure S24. Band structures and density of states of (a) BP, (b) BPEr, (c) SNO, and (d) SNOEr.

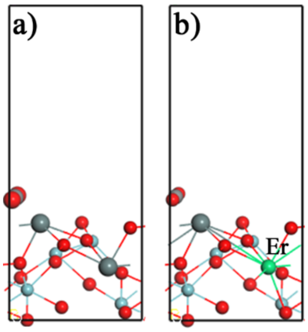

Figure S25. The CO_2 adsorption sites of (a) SNO and (b)SNOEr.
